# 

**Published:** 1857-02

**Authors:** 


### CONTENTS.

| | Page. |
|---|---|
| **Original Communications—** | |
| Annual Address to the Æsculapian Medical Society, delivered October 30th, 1856. By H. R. Payne, M.D. | 55 |
| Scalp-Wound, followed by Purulent Arachnitis—Trephining—Death—Autopsy. By Geo. Amerman, M.D. | 68 |
| Case of Labor Complicated with Prolapsus of the Vagina. By L. C. White, M.D. | 76 |
| **Book Notices—** | |
| Introductory Lecture, delivered at the Opening of the New Orleans School of Medicine, on the 17th Nov. 1856. By E. D. Fenner, M.D. | 78 |
| The Unity of Medicine, an Introductory Lecture, delivered October 14th, 1856. By Alfred Stille, M.D. | 78 |
| The Relations of the Medical to the Legal Profession, being the Introductory Address at the Opening of the Fifty-First Session of the College of Physicians and Surgeons, of New York. By Chandler R. Gilman, M.D. | 78 |
| Proceedings of the American Pharmaceutical Association, at the Fifth Annual Meeting, held in Baltimore, Sept. 1856 | 79 |
| Fifth Biennial Report of the Board of Trustees of the Illinois State Hospital for the Insane. Dec. 1856 | 79 |
| Transactions of the American Medical Association, Vol. IX. 1856 | 83, 90 |
| Cause of Yellow Fever | 86 |
| **Medical Intelligence—** | |
| Proceedings of the Quarterly Meeting of the De Witt County Medical Society, held at Mt. Pleasant, Ill. Jan. 5th, 1857 | 95 |
| **Editorial—** | |
| Tincture of Veratrum Viride—Glycerine in Phthisis | 97 |
| Prurigo—Mortality of Chicago, for 1856 | 98 |
| Medical Journals—Dr. Paris | 99 |

